# A potent and selective small molecule inhibitor of sirtuin 1 promotes differentiation of pluripotent P19 cells into functional neurons

**DOI:** 10.1038/srep34324

**Published:** 2016-09-29

**Authors:** Beom Seok Kim, Chang-Hee Lee, Gyeong-Eon Chang, Eunji Cheong, Injae Shin

**Affiliations:** 1 National Creative Research Initiative Center for Biofunctional Molecules, Department of Chemistry, Yonsei University, Seoul 03722, Korea; 2 Department of Biotechnology, College of Life Science and Biotechnology, Yonsei University, Seoul 03722, Korea

## Abstract

Sirtuin 1 (SIRT1) is known to suppress differentiation of pluripotent/multipotent cells and neural progenitor cells into neurons by blocking activation of transcription factors critical for neurogenesis. EX-527 is a highly selective and potent inhibitor against SIRT1 and has been used as a chemical probe that modulates SIRT1-associated biological processes. However, the effect of EX-527 on neuronal differentiation in pluripotent cells has not been well elucidated. Here, we report an examination of EX-527 effects on neurogenesis of pluripotent P19 cells. The results showed that EX-527 greatly accelerated differentiation of P19 cells into neurons without generation of cardiac cells and astrocytes. Importantly, neurons derived from P19 cells treated with EX-527 generated voltage-dependent sodium currents and depolarization-induced action potentials. The findings indicate that the differentiated cells have electrophysiological properties. The present study suggests that the selective SIRT1 inhibitor could have the potential of being employed as a chemical inducer to generate functionally active neurons.

Neurodegenerative disorders are caused by the progressive loss of structure and function of specific populations of neurons, which leads to irreversible deterioration of the nervous system. These diseases will become progressively more severe in the future because of the increase in the life span of humans. As a consequence, it is of great importance to develop strategies to promote neuronal differentiation of progenitors and multipotent/pluripotent stem cells. The fate of progenitors and stem cells can be modulated by pharmacological intervention with small molecules (termed ‘small molecule-based cellular alchemy’)^1^,^2^,^3^,^4^,^5^. Although a number of studies have been conducted to uncover small molecules that accelerate differentiation of progenitors and stem cells into neurons^6^,^7^,^8^,^9^,^10^,^11^, more effort is needed to develop substances that efficiently induce differentiation processes that generate neurons with physiological functions. Neurogenesis inducers of this type can be used as chemical probes to understand molecular mechanisms underlying cellular plasticity and neurogenesis as well as therapeutic agents.

Epigenetic enzymes, including histone deacetylases, histone acetyltransferases, histone methyltransferases and DNA methyltransferases, are known to play important roles in neuronal differentiation by regulating expression of neuron-associated genes^12^. Among this group are sirtuins (SIRTs), NAD^+^-dependent protein deacetylases, which catalyze the removal of acetyl groups from lysine residues in histones and transcription factors. These enzymes have diverse biological functions, including DNA repair, cell cycle regulation, chromatin silencing, apoptosis and stem cell regulation^13^,^14^,^15^,^16^. Most of studies of SIRTs have been focused on understanding roles they play in aging and tumorigenesis. Relatively less attention has been given to functions of SIRTs in cell differentiation.

Sirtuin 1 (SIRT1), a mammalian homologue of the yeast histone deacetylase Sir2, is the most widely studied member of this enzyme family. SIRT1 is involved in a variety of biological processes, including life-span extension, metabolism, cellular senescence and cancer^17^,^18^,^19^. The role of SIRT1 in cancers has been extensively studied over the last decade^20^,^21^,^22^. Interestingly, SIRT1 is also known to be implicated in self-renewal and maintenance of multipotency/pluripotency, and determining the fate of stem cells and neural progenitor cells^23^,^24^,^25^. In this context, a rapidly growing interest has occurred in examining the role that SIRT1 plays in stem cell differentiation^26^,^27^,^28^,^29^,^30^. SIRT1 acts as a transcriptional co-repressor together with Hes1, which is crucial for stem cell maintenance and prevention of neurogenesis by suppressing activation of the transcription factor Mash1 that is important for neuronal differentiation^30^,^31^. The possibility has been suggested that SIRT1 has a distinct effect on differentiation under different conditions and in different cells^28^,^29^,^32^. Therefore, great interest exists in carrying out investigations aimed at determining whether SIRT1 inhibitors promote differentiation of pluripotent cells into neurons and, in particular, those with physiological functions.

Several small molecule inhibitors of SIRT1 have been exploited for use in understanding SIRT1 associated biological processes. EX-527 (Fig. 1a) is a highly potent and selective small molecule inhibitor against SIRT1 and exhibits much lower or no inhibitory activity against other SIRTs^33^. This inhibitor has been used as a chemical probe that modulates SIRT1 mediated biological events *in vitro* and *in vivo*. For example, EX-527 induces apoptosis in acute myeloid leukemia by inhibiting deacetylation of p53^34^. In addition, EX-527 treatment leads to an increase in levels of acetylated p53 in cells subjected to genotoxic agents^35^. It has been also shown that EX-527 adversely affects early developmental events of vertebrate embryos^36^. Interestingly, EX-527 suppresses deacetylation of mutant huntingtin protein and thus promotes degradation of the acetylated mutant by autophagy^37^. Recently, EX-527 passed Phase II clinical trials to treat Huntington’s disease and thus has great potential as a therapeutic agent^38^.

Despite the previous effort, the effect of EX-527 on neuronal differentiation in pluripotent cells has not been well elucidated. In the study described below, we evaluated the ability of EX-527 to induce differentiation of pluripotent P19 cells into functional neurons. Our results provide evidence that treatment of P19 cells with EX-527 leads to selective and efficient generation of neurons that have electrophysiological activities.

## Results

### EX-527 enhances neuronal differentiation of P19 cells

P19 cells derived from a teratocarcinoma induced in C3H/HeHa mice are known to be pluripotent in their ability to differentiate into various cell types, including neuronal, glial, cardiac and skeletal muscle cells, under different specific conditions^39^,^40^,^41^,^42^. For example, it has been revealed that treatment of P19 cells with dimethyl sulfoxide or 5-azacytidine leads to differentiation into cardiac cells^43^. In addition, retinoic acid has been found to induce differentiation of P19 cells into neuronal and glial cells^42^. Importantly, P19 cells are converted to neurons that possess functional synapses and can be integrated into the brain^44^. As a result, P19 cells were employed in this investigation to determine whether EX-527 induced differentiation of pluripotent cells into physiologically active neurons.

**Figure 1:**
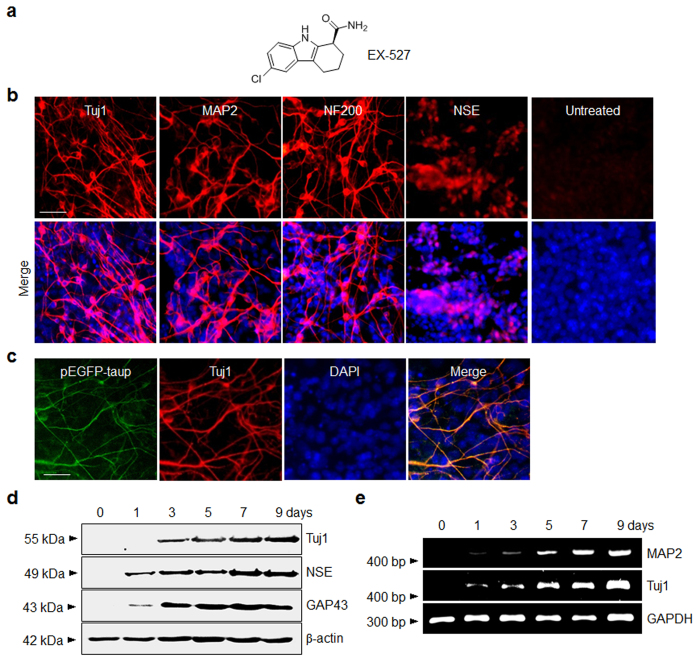
EX-527 promotes neuronal differentiation in P19 cells. (**a**) Chemical structure of EX-527. (**b**) P19 cells were incubated with 100 μM EX-527 for 9 days. (Upper panel) The treated cells were immunostained with antibodies against neuron-specific proteins. (Lower panel) Merged images of cells treated with antibodies and DAPI (blue). ‘Untreated’ indicates no treatment of P19 cells with EX-527. Scale bar, 50 μm. (**c**) P19 cells stably transfected with a Tau promoter-EGFP fusion gene (pEGFP-taup) were incubated with 100 μM EX-527 for 9 days. The treated cells were immunostained with anti-Tuj1 antibody (scale bar; 50 μm). EGFP (green) and Tuj1 (red) colocalize in the treated P19 cells. (**d**) The expression levels of neuron-specific markers in P19 cells treated with 100 μM EX-527 were examined at various times by using western blot and (**e**) RT-PCR analyses. β-Actin and GAPDH were used as loading controls.

In an initial study designed to evaluate the ability of EX-527 to enhance neurogenesis in pluripotent cells, P19 cells were incubated with this SIRT1 inhibitor for 9 days, along with retinoic acid as a control which is a well-known neurogenic inducer^42^. Development of a neuronal phenotype of the treated cells was initially examined by using immunocytochemical analysis with several antibodies against neuron-specific markers, including Tuj1 (neuron-specific class III β-tubulin), microtubule-associated protein 2 (MAP2), neurofilament 200 (NF200) and neuron-specific enolase (NSE). The results showed that P19 cells treated with EX-527 are differentiated into neurogenic cells with a 40–50% efficiency (Fig. 1b), which is comparable to that of retinoic acid (50–60% neurogenesis efficiency) (Figure S1). In contrast, untreated P19 cells seldom differentiate into neurogenic cells. Neurogenesis of P19 cells induced by EX-527 was further demonstrated by examining the Tau promoter activity of the treated cells. In this study, P19 cells stably transfected with a Tau promoter-EGFP fusion gene (pEGFP-taup) were treated with EX-527 and the Tau promoter expression pattern of the treated P19 cells was then determined. The results showed that the level of GFP-positive cells, co-stained with a Tuj1 antibody, increases after treatment with EX-527 (Fig. 1c). This finding provides additional evidence to support the conclusion that EX-527 has the ability to induce neuronal differentiation of P19 cells.

The neurogenesis inducing activity of EX-527 was also examined by using western blot analysis of neuron-specific markers. Consistent with the results obtained using immunocytochemical analysis, EX-527 treatment of P19 cells leads to an increase in expression levels of neuronal markers (Fig. 1d). Furthermore, RT-PCR analysis revealed that mRNAs of neuronal marker genes are upregulated in P19 cells after treatment with EX-527 (Fig. 1e). The results of RT-PCR analysis also showed that key genes related to stemness (Oct4, Nanog, Fgf4, Fgf5, Utf1 and Sox2)^45^ are downregulated in P19 cells treated with EX-527 (Figure S2). The findings demonstrate that because SIRT1 is involved in self-renewal and maintenance of pluripotency/multipotency, its inhibition by EX-527 leads to abrogation of stemness of P19 cells and subsequent induction of neuronal differentiation.

**Figure 2:**
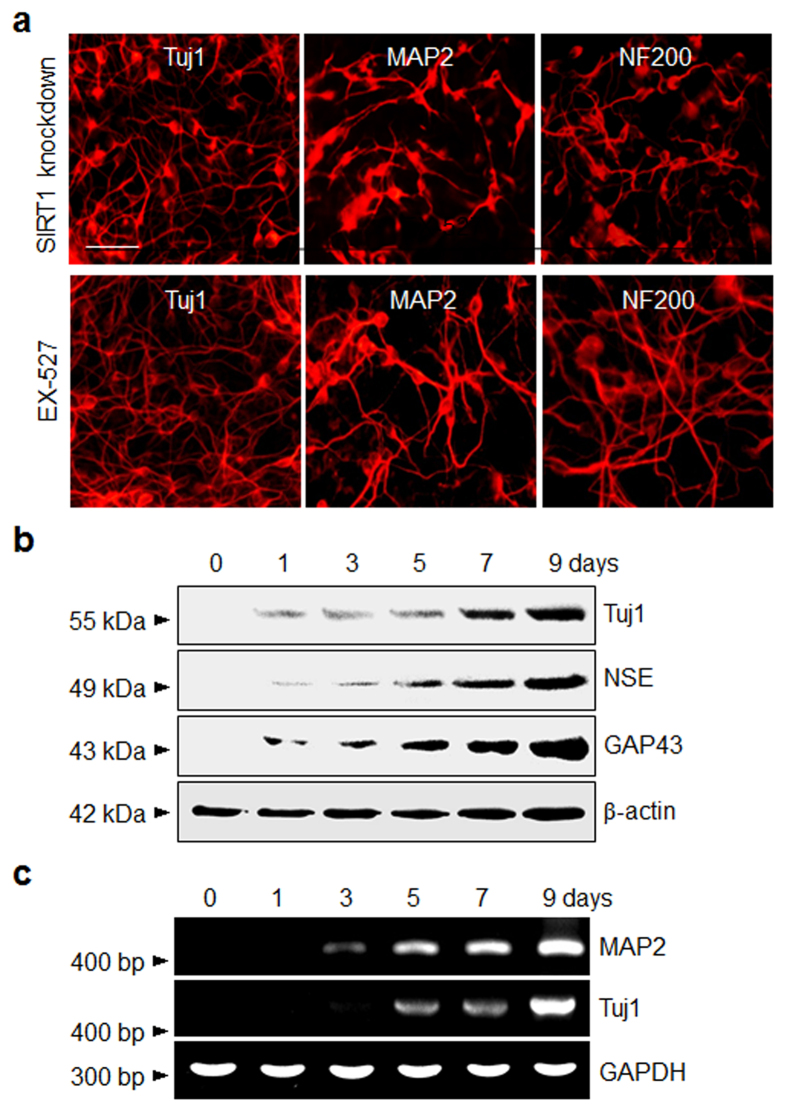
Knockdown of SIRT1 promotes neurogenesis in P19 cells. (**a**) (Upper panel) SIRT1 knockdown P19 cells were cultured for 9 days without any treatment. (Lower panel) P19 cells were treated with 100 μM EX-527 for 9 days. The SIRT1 knockdown cells and EX-527 treated cells were immunostained with antibodies against neuron-specific proteins (scale bar; 50 μm). (**b**) The expression levels of neuron-specific markers in SIRT1 knockdown P19 cells were determined at various times by using western blot and (**c**) RT-PCR analyses.

Additional evidence supporting the conclusion that inhibition of SIRT1 induces neurogenesis of P19 cells came from knockdown experiments. For this purpose, SIRT1 knockdown P19 cells (Figure S3) were cultured for 9 days without any treatment^46^. The results of immunostaining and RT-PCR analyses revealed that knockdown of SIRT1 enhances neuronal differentiation in P19 cells (Fig. 2)^47^, a phenomenon which was observed in cells treated with EX-527. Next, to examine the effect of a SIRT1 activator on neurogenesis, P19 cells were exposed to the well-known SIRT1 activator, resveratrol, for 9 days^48^. As the results in Figure S4 show, resveratrol treatment does not induce neurogenesis of P19 cells, as inferred from the lack of immunostaining with antibodies against neuronal markers. Taken together, the findings demonstrate that SIRT1 plays a role in suppressing the neuronal fate specification of pluripotent P19 cells and that its inhibitor EX-527 enhances the development of a neuronal phenotype.

### P19 cells are not converted to astrocytes or cardiac cells after treatment with EX-527

**Figure 3:**
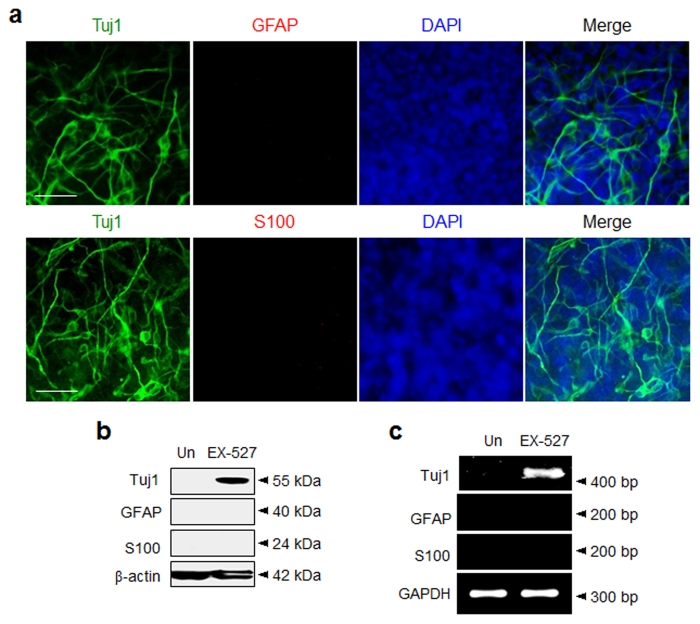
P19 cells treated with EX-527 are differentiated into neurons but not astrocytes. (**a**) P19 cells were incubated with 100 μM EX-527 for 9 days and then immunostained with antibodies against neuronal (Tuj1, green) and astrocyte-specific markers (upper: GFAP and lower: S100, red). The nucleus of the cells was stained with DAPI (blue) (scale bar: 50 μm). (**b**) The expression levels of astrocyte markers in P19 cells treated with EX-527 for 9 days were examined by using western blot and (**c**) RT-PCR analyses. ‘Un’ indicates no treatment of P19 cells with EX-527.

The effect of EX-527 on lineage-specific differentiation of P19 cells was investigated. It is well recognized that P19 cells are differentiated into neurons as well as astrocytes by treatment with retinoic acid^42^. This finding led us to question whether EX-527 treatment stimulates differentiation of P19 cells into astrocytes. To obtain information about this issue, expression levels of astrocyte markers, such as glial fibrillary acidic protein (GFAP) and S100, were measured in P19 cells treated with EX-527, along with retinoic acid as a control. The results of immunocytochemical analysis showed that in contrast to P19 cells treated with retinoic acid that express high levels of GFAP and S100 (Figure S5), cells exposed to EX-527 seldom produce astrocyte positive cells (Fig. 3a and also see Figure S7b). The astrocyte-suppression activity of EX-527 was further demonstrated by using western blot and RT-PCR analyses of astrocyte markers. Consistently, in comparison to retinoic acid (Figure S5b,c), EX-527 treatment does not result in an increase in expression levels of astrocyte markers in cells (Fig. 3b,c and also see Figure S7c).

**Figure 4:**
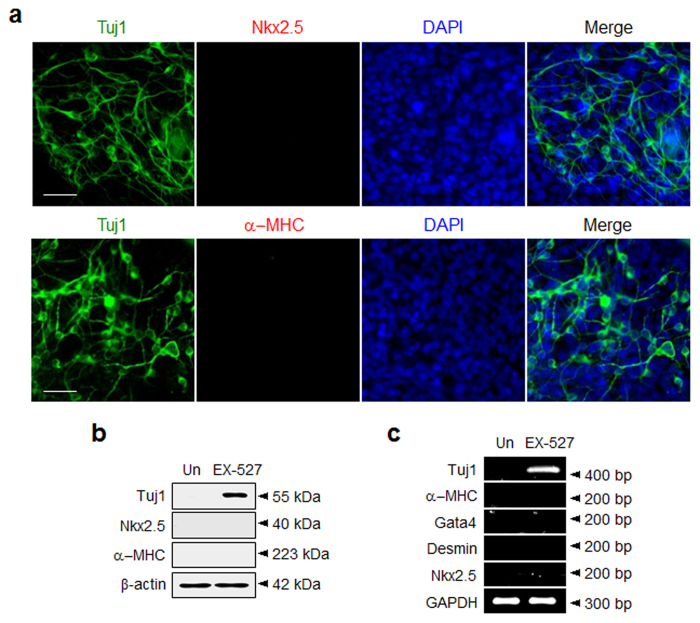
P19 cells treated with EX-527 are differentiated into neurons but not cardiac cells. (**a**) P19 cells were incubated with 100 μM EX-527 for 14 days. The cells were immunostained with antibodies against neuronal (Tuj1, green) and cardiac markers (upper: Nkx2.5 and lower: α-MHC, red) (scale bar: 50 μm). The nucleus of the cells was stained with DAPI (blue). (**b**) The expression levels of cardiac markers in the treated P19 cells for 14 days were examined by using western blot and (**c**) RT-PCR analyses. ‘Un’ indicates no treatment of P19 cells with EX-527.

It is also known that P19 cells have the ability to differentiate into cardiac cells^48^. Thus, the ability of EX-527 to induce cardiogenesis of P19 cells was explored. For this purpose, P19 cells were incubated for 14 days with EX-527, along with 5-azacytidine, a known inducer of cardiogenesis in P19 cells^48^. Expression levels of cardiac marker proteins, such as α−myosin heavy chain (α−MHC) and Nkx2.5, were then determined. As shown in Fig. 4a, EX-527 treatment does not induce cardiogenesis but only elicits neurogenesis in P19 cells, as demonstrated by the observations of positive immunostaining with an antibody against a neuronal marker (Tuj1) and negative immunostaining with antibodies against cardiac markers (α−MHC and Nkx2.5). These observations are the opposite of those arising from experiments with 5-azacytidine, which showed that the treated cells are differentiated into cardiac cells and are not converted to neurogenic cells (Figure S6). The absence of EX-527 promoted differentiation of P19 cells into cardiac cells gains further support from the results of western blot and RT-PCR analyses (Fig. 4b,c). In addition, we shortly examined the ability of nicotinamide, which is less selective and less potent SIRT1 inhibitor than EX-527, to induce neuronal differentiation of P19 cells. As shown in Figure S7, nicotinamide enhances neuronal differentiation of P19 cells without generation of astrocytes.

It is known that during Notch signaling, the Notch intracellular domain is liberated into the cytosol and subsequently it translocates to the nucleus to activate the transcription of Hes1, which acts as a repressor to block expression of neuronal genes (*e.g.* Mash1 and neurogenin-2 (Neurog2))^32^,^49^. This cascade leads to maintenance of neural stem cells in an undifferentiated state. In this signaling pathway SIRT1 plays a role in upregulating Hes1 and blocking activation of transcription factors Mash1 and Neurog2 which are important for the acquisition of neuronal properties^50^, thereby leading to suppression of neurogenesis.

**Figure 5:**
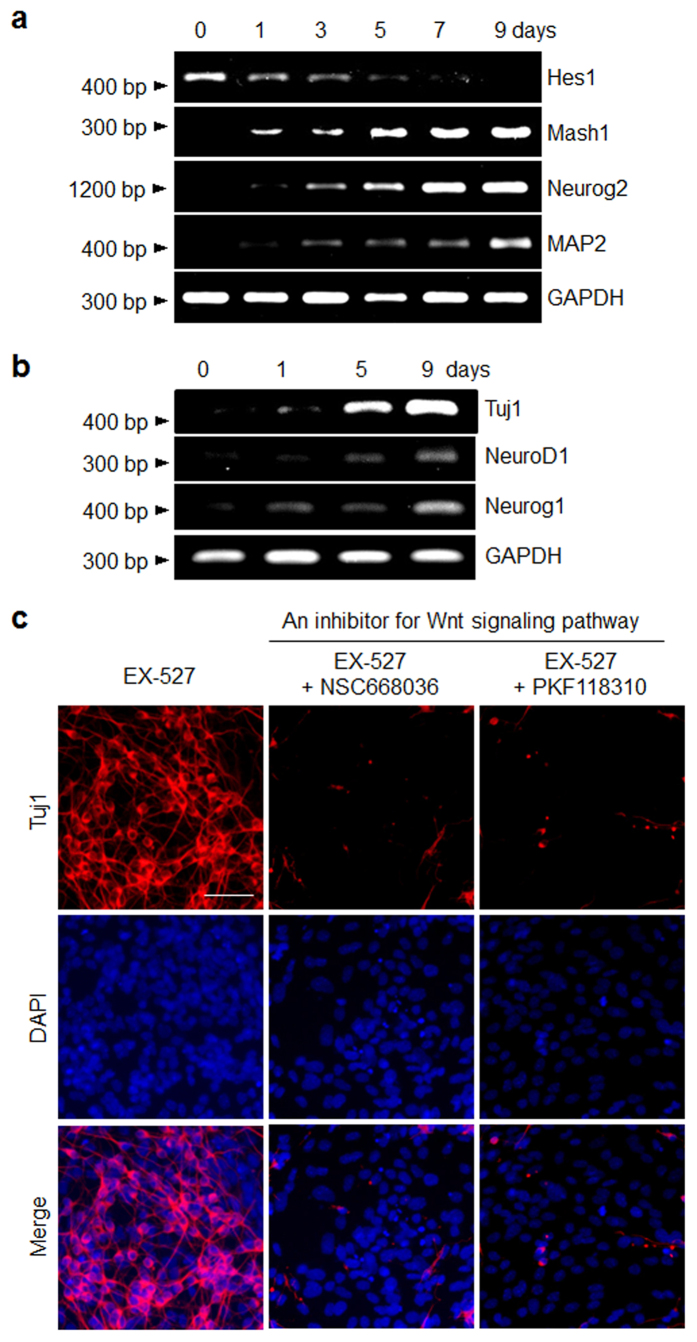
Signaling pathways associated with EX-527 mediated neurogenesis of P19 cells. P19 cells were incubated with 100 μM EX-527 for the indicated times. (**a**) The transcriptional levels of genes associated with Notch and (**b**) Wnt signaling pathways were examined by using RT-PCR analysis. (**c**) P19 cells were incubated for 9 days with 100 μM EX-527 in the absence (left) and presence of an inhibitor for the Wnt signaling pathway, 25 μM NSC668036 (middle) or 25 nM PFK118-310 (right). The cells were immunostained with Tuj1 antibody. Merged images include DAPI to illustrate nuclei (scale bar: 50 μm).

With these considerations in mind, we examined mRNA levels of Hes1, Mash1 and Neurog2 in P19 cells after treatment with EX-527 by using RT-PCR analysis. The results showed that while the expression level of Hes1 in the cells is gradually attenuated by treatment with EX-527, the mRNA levels of Mash1 and Neurog2 as well as neuronal markers increase significantly (Fig. 5a). This finding suggests that inhibition of SIRT1 in P19 cells by EX-527 causes Hes1 to be downregulated and transcription of Mash1 and Neurog2 to be activated in order to stimulate neuronal differentiation of the pluripotent cells.

We also examined the effect of EX-527 on the Wnt signaling pathway, which is a key regulator of neuronal differentiation^51^. In this study, the effects of EX-527 on expression of target genes (NeuroD1 and neurogenin-1 (Neurog1)) of the Wnt signaling pathway were determined^52^. P19 cells were exposed to EX-527 for different time periods and then subjected to RT-PCR. The results showed that expression levels of NeuroD1 and Neurog1 increase after EX-527 treatment (Fig. 5b), suggesting that the Wnt signaling pathway may be activated during EX-527 promoted neurogenesis of P19 cells. To gain support for this conclusion, P19 cells were incubated with EX-527 in the presence and absence of the Wnt pathway inhibitors, NSC668036 and PKF118-310. NSC668036 inhibits the Wnt pathway by binding to the PDZ domain of the Disheveled (Dsh) protein^53^, and PKF118-310 suppresses the pathway by blocking complex formation between Tcf4 and β-catenin^54^. The results of immunocytochemical analysis of the treated P19 cells indicated that neurogenesis is greatly abrogated in the presence of each of NSC668036 and PKF118-310 as compared to that of cells that are not treated with an inhibitor (Fig. 5c). Collectively, the results of the signaling pathway studies suggest that treatment with EX-527 accelerates neurogenesis in P19 cells presumably by suppressing the Notch signaling pathway and activating the Wnt pathway.

### Expression of neurotransmitter receptors in EX-527 treated P19 cells

A variety of neurotransmitter receptors are distinctively produced during neuronal differentiation. Among these are glutamate receptors, which are associated with the glutamate-mediated postsynaptic excitation of neurons and which play important roles in neural communication, memory formation and learning^55^. These receptors are classified into ionotropic and metabotropic glutamate receptors based on the mechanism they utilize for activation^56^. Specifically, ionotropic glutamate receptors form ion channel pores and are excited upon binding of glutamate whereas metabotropic glutamate receptors (mGluRs) indirectly activate ion channels located on plasma membranes through G protein associated signaling pathways.

A study was conducted to determine if neurogenic cells derived from treatment of P19 cells with EX-527 express glutamate receptors. RT-PCR analysis of cells exposed to EX-527 showed that EX-527 treatment increases expression levels of the ionotropic glutamate receptors, GluA2, GluA3 and GluK5, as well as the metabotropic glutamate receptor, mGluR7 (Figure S8). These observations indicate that certain isoforms of glutamate receptors are expressed during EX-527 promoted neurogenesis of P19 cells.

### SIRT1 promotes differentiation of P19 cells into electrophysiologically active neurons

Exploitation of neurogenic inducers, which have the ability to promote differentiation of cells into functional neurons, is an important challenge in clinical neuroscience. As a result, we examined whether neurons derived by treating P19 cells with EX-527 display electrophysiological activities. Because voltage-gated sodium channels are known to be crucial for generating the action potential and electrical excitability of neurons^57^, the expression levels of these channels in treated P19 cells were initially measured by using RT-PCR analysis. The results showed that mRNA levels of several sodium channels, including SCN1A, SCN2A1, SCN3A and SCN8A, are increased in differentiated P19 cells after treatment with EX-527 (Figure S9).

**Figure 6:**
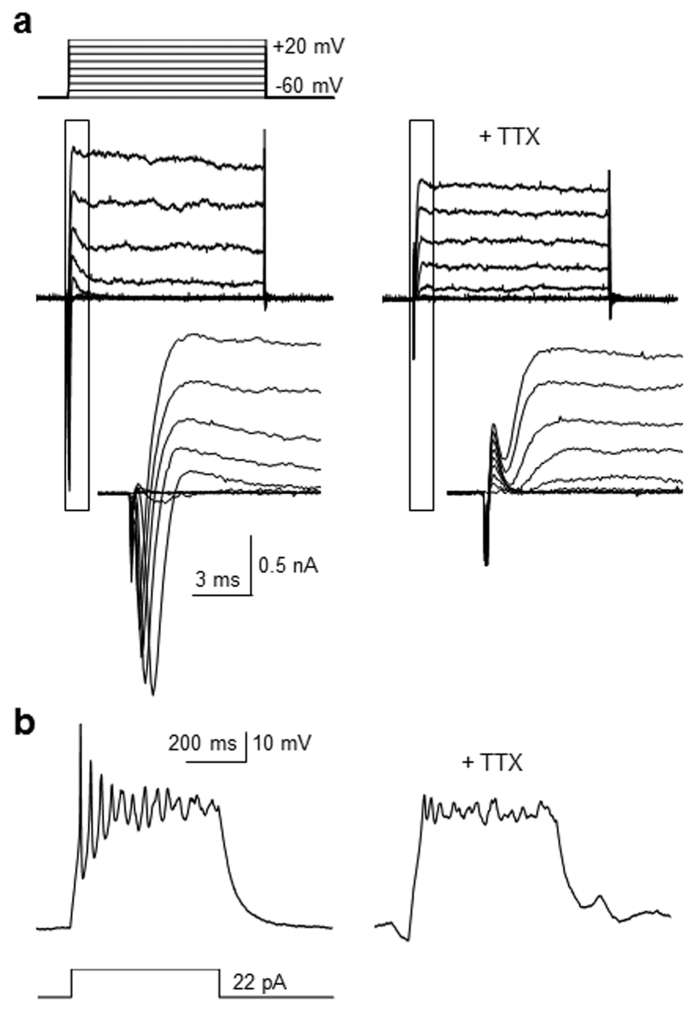
Neurons derived from P19 cells treated with EX-527 have electrophysiological activities. (**a**) P19 cells were incubated with 100 μM EX-527 for 14 days. Whole-cell currents in the differentiated cells were measured by depolarizing voltage ranged from −60 mV to +20 mV, with a 10-mV step before (left) and after (right) 0.5 μM TTX perfusion (n = 6/10 of recorded cells). The scales of time and current are presented. Insets show respective traces on an expanded scale. (**b**) P19 cells were incubated with 100 μM EX-527 for 14 days. Action potentials in the differentiated cells were recorded in response to depolarization by current injection before (left) and after (right) 0.5 μM TTX perfusion (n = 6/10 of recorded cells). The scales of time and voltage are presented.

Encouraged by these findings, the electrophysiological activities of neurogenic cells, generated by treatment with EX-527, were determined. Towards this end, P19 cells were exposed to EX-527 and whole-cell patch-clamp analyses were performed on the differentiated cells. It was found that cells with a neuronal phenotype have an average resting membrane potential of −60 mV in response to depolarizing current pulses. Whole-cell currents induced by 10 mV depolarizing voltage steps from −60 mV to +20 mV were then measured in the voltage-clamp mode. Fast-inactivating inward and outward currents were observed in *ca.* 60% of the recorded cells (Fig. 6a). In marked contrast, addition of tetrodotoxin (TTX), a selective blocker of sodium channels^58^, causes the treated cells not to display depolarization-induced currents. The findings indicate that the recorded current in the differentiated P19 cells is sodium channel dependent.

Next, to determine the capability of generating depolarization-induced action potentials, the neurogenic cells derived from EX-527 treated P19 cells were subjected to current clamp measurements. The results showed that relatively high amplitudes of action potentials are elicited in differentiated P19 cells by current injection, a phenomenon that is almost completely abolished in the presence of TTX (Fig. 6b). Collectively, the observations provide evidence to support the conclusion that EX-527 induces differentiation of P19 cells into electrophysiologically active neurons.

## Discussion

SIRT1 is known to suppress the activation of neuron-associated genes critical for neuronal differentiation, thereby blocking neuronal differentiation. On this basis, here we investigated the ability of a small molecule SIRT1 inhibitor to promote neuronal differentiation of pluripotent cells. We found that a highly selective and potent SIRT1 inhibitor, EX-527, enhanced neuronal differentiation of pluripotent P19 cells. In addition, knockdown of SIRT1 in P19 cells led to the promotion of neurogenesis. Our findings are consistent with the results of previous studies showing that SIRT1 knockdown induces differentiation of neural progenitor cells^29^,^47^ P19 cells^46^ and neuro2a cells^59^ into neuron-like cells.

Because SIRT1 acts as a transcriptional repressor to keep stemness and pluripotency of stem cells, it is speculated that inhibition of SIRT1 would abrogate the pluripotency of P19 cells and direct differentiation of P19 cells into certain cell types. Our results showed that treatment of P19 cells with EX-527 led to downregulation of several stemness-associated genes and upregulation of neuronal genes. The findings support the notion that SIRT1 plays a role in self-renewal and maintenance of pluripotency and its inhibition leads to promotion of neurogenesis in P19 cells. Importantly, EX-527 induced selective neuronal differentiation in P19 cells because cardiogenesis and astrogenesis were suppressed. However, treatment of P19 cells with a well-known neurogenic inducer, retinoic acid, promoted both neurogenesis and astrogenesis, indicating that EX-527 is a more selective neurogenesis-inducing agent than retinoic acid. Moreover, we examined the effect of another SIRT1 inhibitor, nicotinamide, on differentiation of P19 cells. We found that nicotinamide had neurogenesis-inducing activity in P19 cells like EX-527. Previously, it has been shown that nicotinamide, which is a less slective and less potent inhibitor for SIRT1 than EX-527^60^,^61^, suppresses neurogenesis of neuronal progenitor cells^28^ but stimulates neuronal differentiation of mouse embryonic stem cells^62^. We surmise that the controversial effect of nicotinamide on neurogenesis could result from less selective inhibitory activity against SIRT1, different cell types and/or different experimental conditions used for differentiation.

Additionally, we investigated the effect of EX-527 on Notch and Wnt signaling pathways associated with neurogenesis of P19 cells. Firstly, it is likely that EX-527 induces neurogenesis by suppressing the Notch pathway, as inferred from observations that expression of a repressor Hes1 is downregulated, while expression of Mash1 and Neurog2, which are important transcription factors for neurogenesis, are upregulated in the treated cells. Secondly, EX-527 mediated neurogenesis may integrate the Wnt signaling pathway because the expression levels of two Wnt signaling target genes, such as NeuroD1 and Neurog1, were increased in the treated P19 cells, and neurogenesis of P19 cells by EX-527 was greatly attenuated in the presence of each of upstream (NSC668036) and downstream (PKF118-310) inhibitor for the Wnt pathway. The findings suggest that Wnt and Notch signaling pathways may be involved in EX-527 promoted neuronal differentiation of P19 cells.

It is of great importance to uncover neurogenic inducers which have the ability to induce differentiation of cells into functional neurons for therapeutic applications. We demonstrated that the expression levels of voltage-gated sodium channels critical for generating the action potential and electrical excitability of neurons were increased in P19 cells treated with EX-527. Importantly, the results of whole-cell patch-clamp analysis revealed that the differentiated P19 cells by EX-527 generated voltage-dependent inward and outward sodium currents and depolarization-induced action potentials. This finding indicates that P19 cells treated with EX-527 are differentiated into electrophysiologically active neurons. Collectively, we envision that the selective and potent small molecule SIRT1 inhibitor, EX-527, may be a useful chemical inducer to produce functionally active neurons from pluripotent P19 cells. In addition, this inhibitor has great potential for use in understanding the molecular mechanisms underlying cellular neurogenesis.

## Methods

### Cell culture

Mouse P19 embryonic carcinoma cells (ATCC) were maintained at a relatively high density in culture media (RPMI 1640 media supplemented with 10% fetal bovine serum (FBS) and 50 units/ml penicillin and 50 μg/ml streptomycin). The cells were grown to confluence in a cell culture incubator (5% CO_2_) at 37 ^o^C in 100 cm^2^ tissue culture dishes. The cells were sub-cultured after they formed a confluent monolayer (approximately 24–48 h).

### Differentiation of P19 cells by EX-527

For neurogenesis and astrogenesis study, P19 cells were seeded at a density of 10^6 ^cells/ml in 90 mm petri dishes under non-adherent culture conditions and then incubated with 1 μM retinoic acid in a cell culture incubator for 3 days. The embryoid bodies were dissociated into single cells by treatment with 0.25% trypsin-EDTA solution. After washing cells with Dulbecco’s phosphate buffered saline (DPBS) to remove remaining retinoic acid, trypsin and EDTA, they were seeded in a tissue culture dish at a density of approximately 10^5 ^cells/ml in culture media. After incubation for 24 h, the culture media were replaced with differentiation media (RPMI 1640 supplemented with 2% FBS, 50 units/ml penicillin, 50 mg/ml streptomycin and a small molecule (100 μM EX-527, 10 mM nicotinamide or 1 μM retinoic acid (a positive control)). Differentiation media were replenished every 2 days. Cells were harvested and analyzed during differentiation.

For cardiogenesis study, P19 cells were seeded at a density of 10^6 ^cells/mL in 90 mm petri dishes under non-adherent culture conditions and then incubated with 1 μM 5-azacytidine in a cell culture incubator for 4 days. The embryoid bodies were dissociated into single cells by treatment with 0.25% trypsin-EDTA solution. The cells were seeded in a tissue culture dish at a density of approximately 10^5 ^cells/mL in culture media. After incubation for 24 h, the culture media were replaced with differentiation media (RPMI 1640 supplemented with 2% FBS, 50 units/ml penicillin, 50 mg/ml streptomycin and a small molecule (100 μM EX-527 or 1 μM 5-azacytidine (a positive control)). Differentiation media were replenished every 2 days. Cells were harvested and analyzed after differentiation.

### Electrophysiological study

After differentiation of P19 cells by treatment with 100 μM EX-527 for 14 days, cells with neuronal morphology (round cell body and neurite-like processes) were selected for whole-cell patch clamp recordings. Cells plated on coverslips were placed in a submerged recording chamber (Warner Instruments) on a microscope (Olympus). Electrophysiological recordings were made using a Multiclamp 700B amplifier (Molecular Devices) and Clampex 10.3 of the pClamp software package (Molecular Devices). Digitization of voltages and currents was controlled by Digitizer 1440A (Molecular Devices). Recording electrodes were pulled from borosilicate capillary glass tubes (Warner Instruments) using a pipet puller (P-97, Sutter Instrument). Resistance of recording electrodes ranged from 4 MU to 6 MU when they were filled with an intra-pipette solution (115 mM potassium gluconate, 10 mM KCl, 10 mM HEPES, 10 mM EGTA, 5 mM Mg^2+^-ATP, and 0.5 mM 2Na^+^ -GTP [pH 7.3], and 280–285 mOsm). Artificial cerebrospinal fluid was used as an external solution (124 mM NaCl, 3 mM KCl, 1.3 mM MgSO_4_, 1.25 mM NaH_2_PO_4_, 26 mM NaHCO_3_, 10 mM glucose, and 2.4 mM CaCl_2_·2H_2_O). The external solution was aerated with a mixed gas of 95% O_2_ and 5% CO_2_ at room temperature. During the whole-cell configuration, the holding potential was clamped to −60 mV in a voltage-clamp mode. To measure the sodium current, the cell was stimulated in 100-ms voltage steps of depolarization from the −60 mV holding potential to +20 mV (+10 mV per each step). In a current-clamp mode, the cells were subjected to a series of current injections to examine generation of action potentials. Tetrodotoxin (0.5 μM TTX, Sigma-Aldrich) was applied to the external solution to confirm currents and action potentials elicited by Na_v_ channels. After perfusion with 0.5 μM TTX in an external solution for 5–10 min, currents and action potentials were measured by following the above experimental process.

## Additional Information

**How to cite this article**: Kim, B. S. *et al*. A potent and selective small molecule inhibitor of sirtuin 1 promotes differentiation of pluripotent P19 cells into functional neurons. *Sci. Rep.* **6**, 34324; doi: 10.1038/srep34324 (2016).

## Supplementary Material

Supplementary Information
