# Supplementary material for: A potent and selective small molecule inhibitor of sirtuin 1 promotes differentiation of pluripotent P19 cells into functional neurons
Source: Sci Rep. 2016 Sep 29;6:34324. doi: 10.1038/srep34324 (PMC5041152; doi:10.1038/srep34324)
Supplement: Supplementary Information [file srep34324-s1.pdf]

SUPPLEMENTARY INFORMATION

A potent and selective small molecule inhibitor of sirtuin 1 promotes differentiation of pluripotent P19 cells into functional neurons

Beom Seok Kim,[1] Chang-Hee Lee,[1] Gyeong-Eon Chang,[2] Eunji Cheong,[2] and Injae Shin,[1,*]

[1]National Creative Research Initiative Center for Biofunctional Molecules, Department of Chemistry, Yonsei University, Seoul 03722, Korea

[2]Department of Biotechnology, College of Life Science and Biotechnology, Yonsei University, Seoul 03722, Korea

* Corresponding author: injae@yonsei.ac.kr

Methods

Differentiation of SIRT1 knockdown P19 cells. SIRT1 knockdown P19 cells were constructed by transfection with a SIRT1 shRNA plasmid (Santa Cruz) using plasmid transfection reagents (Santa Cruz) according to the manufacturer's instruction. Shortly, P19 cells were seeded at approximately 2 x 10^5 per well in a six-well dish, grown to 50–70% confluency, and transfected with 1 μg of a SIRT1 shRNA plasmid for 24 h. Stably transfected P19 cells were selected by incubating with 8 μg/ml of puromycin (Sigma-Aldrich) for a minimum of 3 weeks.

SIRT1 knockdown P19 cells were seeded at a density of 10^5 cells/mL in 90 mm petri dishes under non-adherent culture conditions and allowed for aggregation for 3 days. The embryoid bodies were dissociated into single cells by treatment with 0.25% trypsin-EDTA solution. The cells were seeded in a tissue culture dish at a density of approximately 5 x 10^3 cells/mL in culture media. After incubation for 24 h, the culture media were replaced with RPMI 1640 supplemented with 2% FBS, 50 units/ml penicillin and 50 mg/ml streptomycin. The culture media were replenished every 2 days. Cells were harvested and analyzed during differentiation.

Immunocytochemistry. The differentiated P19 cells were fixed for 20 min with 4% paraformaldehyde and 0.1% Triton X-100 in PBS and then washed with PBS. The fixed cells were incubated in blocking solutions (PBS containing 0.5% FBS) for 1 h at room temperature to reduce nonspecific adsorption of antibodies. The cells were treated with diluted primary antibodies in blocking solutions for 1 h at room temperature or overnight at 4 ^{o}C and then washed with PBS. The cells were treated with Alexa Fluor 488-conjugated secondary antibodies or biotin-labeled secondary antibodies followed by incubation with Cy3-conjugated streptavidin in blocking solutions for 1 h at room temperature. Antibodies against neuron-specific βIII-tubulin (Tuj1), neuron specific enolase, neurofilament 200, MAP2, Nkx2.5, α−MHC, GFAP and S100 were employed as primary antibodies (Table S1). Biotin-conjugated goat anti-mouse IgG, goat anti-rabbit IgG, Alexa Fluor 488-conjugated goat anti-mouse IgG and goat anti-rabbit IgG were used as secondary antibodies. After staining, cells were washed with DPBS several times and imaged by using fluorescence microscopy (Nikon Eclipse Ti-U microscope).

Table S1. Antibodies and concentrations used for immunocytochemistry.

	Name	Manufacturer	Clone	Cat. No.	Dilution
Primary antibody	Tuj1	Santa Cruz Biotechnology	Mouse monoclonal Clone TUJ-1	sc-58888	1:200
	NSE	Santa Cruz Biotechnology	Mouse monoclonal Clone NSE-P1	sc-21738	1:200
	NF200	Sigma Aldrich	Rabbit polyclonal	N4142	1:200
	GFAP	Abcam	Rabbit polyclonal	ab7260	1:1000
	S100	Abcam	Rabbit polyclonal	ab868	1:100
	MAP-2	Santa Cruz Biotechnology	Rabbit monoclonal Clone H-300	sc-20172	1:200
	α-MHC	Abcam	Mouse monoclonal Clone BA-G5	ab50967	1:200
	Nkx2.5	Santa Cruz Biotechnology	Mouse monoclonal Clone A-3	sc-376565	1:100
Secondary antibody	Biotin-conjugated Ab	Sigma Aldrich	goat anti-Mouse IgG	B7264	1:250
			goat anti-Rabbit IgG	B7389	1:250
	Alexa Flour 488 conjugated Ab	Thermo Scientific	goat anti-Mouse IgG	A-11001	1:250
			goat anti-Rabbit IgG	A-11034	1:250

Western blot analysis. Proteins were separated by using 10% or 12% SDS-PAGE and transferred to the membrane (Pall Corporation). The membrane was incubated in blocking solutions (Tris-buffered saline (TBS) containing 5% nonfat skim milk and 0.5% Tween 20) for 1-2 h at room temperature to reduce nonspecific adsorption of antibodies. After the membrane was washed with TBST (TBS containing 0.5% Tween 20), it was treated with diluted primary antibodies in TBST for 1 h at room temperature or overnight at 4 ^{o}C. After washing with TBST, it was treated with diluted secondary antibodies in TBST for 1 h at room temperature. The treated membrane was visualized using the West-zol kit (iNtRON Biotechnology) in the G box chemi XT4 (Syngene). Antibodies against Tuj1, neuron specific enolase, GAP43, Nkx2.5, α–MHC, GFAP and S100 were used as primary antibodies (Table S2). Peroxidase-conjugated goat anti-mouse IgG and goat anti-rabbit IgG were employed as secondary antibodies.

Table S2. Antibodies and concentrations used for western blotting.

	Name	Manufacturer	Clone	Cat. No.	Dilution
Primary Antibody	Tuj1	Santa Cruz Biotechnology	Mouse monoclonal Clone TUJ-1	sc-58888	1:1000
	NSE	Santa Cruz Biotechnology	Mouse monoclonal Clone NSE-P1	sc-21738	1:1000
	GAP43	Santa Cruz Biotechnology	Mouse monoclonal Clone B-5	sc-17790	1:1000
	GFAP	Abcam	Rabbit polyclonal	ab7260	1:10000
	S100	Abcam	Rabbit polyclonal	ab868	1:500
	β-actin	Santa Cruz Biotechnology	Mouse monoclonal Clone C4	sc-47778	1:1000
	α-MHC	Abcam	Mouse monoclonal Clone BA-G5	ab50967	1:1000
	Nkx2.5	Santa Cruz Biotechnology	Mouse monoclonal Clone A-3	sc-376565	1:1000
Secondary Antibody	Peroxidase conjugated Antibody	Santa Cruz Biotechnology	goat anti-Mouse IgG	sc-2005	1:1000
			goat anti-Rabbit IgG	sc-2004	1:1000

Signaling pathway study. P19 cells were seeded at a density of 10^6 cells/ml in 90 mm petri dishes under non-adherent culture conditions and then incubated with 0.5 μM retinoic acid in a cell culture incubator for 3 days. The embryoid bodies were dissociated into single cells by treatment with 0.25% trypsin-EDTA solution. After washing with DPBS to remove remaining retinoic acid, trypsin and EDTA, the cells were seeded in a tissue culture dish at a density of approximately 10^5 cells/ml in culture media. After incubation for 24 h, the culture media were replaced with differentiation media (RPMI 1640 supplemented with 2% FBS, 50 units/ml penicillin and 50 mg/ml streptomycin containing 100 μM EX-527) in the presence and absence of an inhibitor for the Wnt signaling pathway (25 μM NSC668036 or 25 nM PKF118-310). Differentiation media without each inhibitor were replenished every 2 days.

Reverse transcription polymerase chain reaction (RT-PCR). Total RNAs were isolated using easy-BLUE (iNtRON Biotechnology) from the differentiated P19 cells at various times and subjected to cDNA synthesis using oligo(dT) primer and power cDNA synthesis kit (iNtRON Biotechnology) according to the manufacturer's instruction. The resulting cDNAs were used to determine the expression levels of genes of interest by using RT-PCR with the

Bio-Rad PCR system. The PCR primers shown in Table S3 were purchased from Bioneer (Korea).

Table S3. Oligonucleotide sequences of primers used for RT-PCR

Target (Mouse)	5’-Forward primer-3’	5’-Reverse primer-3'	Size
NeuroD1	TTGAAGCCATGAATGCAGAG	TTTCAAAGAAGGGCTCCAGA	316
Mash1	TTCGTCCCCCTTTGATCGTG	CCTCCCCATTTGACGTCGTT	293
Neurog2	CAGACAACCACGCACGAGA	CAGGTGAGGCGCATAACGAT	1265
Neurog1	GCGCTTCGCCTACAACTACAT	CGAGGGACTACTGGGGTCAG	417
Hes1	GGTGCTGATAACAGCGGAAT	ATGCCGGGAGCTATCTTTCT	447
Tuj1	CTCCCTTCGATTCCCTGGTC	TGCTCCGAGATGCGTTTGA	439
MAP2	GGATGGGCTTGTGTCTGATT	TGGACCCACTCCACAAACT	428
NF200	AGCCTGCACTACTCGCTGA	GGCCGTTGCTTAGGGTGTC	178
Oct4	AGGGATGGCATACTGTGGAC	CCTGGGAAAGGTGTCCTGTA	752
Nanog	GAGGAAGCATCGAATTCTGG '	AAGTTATGGAGCGGAGCAGC	710
Sox2	CTATTCTCCGCCAGATCTCC	AATCTCTCCCCTTCTCCAGTTC	1143
Nkx2.5	GACAAAGCCGAGACGGATGG	CTGTCGCTTGCACTTGTAGC	222
GATA4	GCAGCAGCAGTGAAGAGATG	GCGATGTCTGAGTGACAGGA	186
α-MHC	TGCACTACGGAAACATGAAGTT	CGATGGAATAGTACACTTGCTGT	204
Desmin	GTGGATGCAGCCACTCTAGC	TTAGCCGCGATGGTCTCATAC	218
S100	AATGTGTTCCATGCCCATTCG	ACCAGCACAACATACTCCTTG	197
GFAP	TCTCGAATGACTCCTCCACTC	AAGCTCCGCCTGGTAGACAT	243
GAPDH	GCCATCAACGACCCCTTCAT	TTCACACCCATCACAAACAT	314
Utf1	CCGTCGCTACAAGTTCCT	AGAGTGTCGGTGCTCGTA	342
Fgf5	AGTGGCTGGGCTCAATGATCAGAA	ATCTCTGTGGACGCTGCACACTTA	121
Fgf4	CTACTGCAACGTGGGCATC	TCGGTAAAGAAAGGCACACC	1201
SCN1a	TCAGAGGGAAGCACAGTAGAC	TTCCACGCTGATTTGACAGCA	138
SCN2a1	TTCATGGCTTCCAATCCCTCC	GGTGTCACGTCAGTCTTCTCT	254
SCN3a	TTGCTGCTATCGAAAAGCGTG	GCACTGAATCGAAAAATTGCCT	247
SCN8a	ACCCGTACTATTTGACGCAGA	TCCCTGTGAATGTGTACTCCA	249
SIRT1	TGACTGGAGCTGGGGTTTCTGT	ATCAGGTAGTTCCTCGGTGCCC	412
mGluR7	AGAGCCCGACCCTAAAAAGAA	CCCTCCTCCCTCTCAATAGC	171
GluK5	TGGCCCTCTTTATCATGGAG	AGGTTGACTAGGCTGCT	469
GluA2	TGATGAACCGCACTGACCCTA	GGAAGAACGTGGATGTCGGA	219
GluA3	CTGGAGGCCATGTTGTTTGC	TGTACGAACCGCCAATGACTC	257

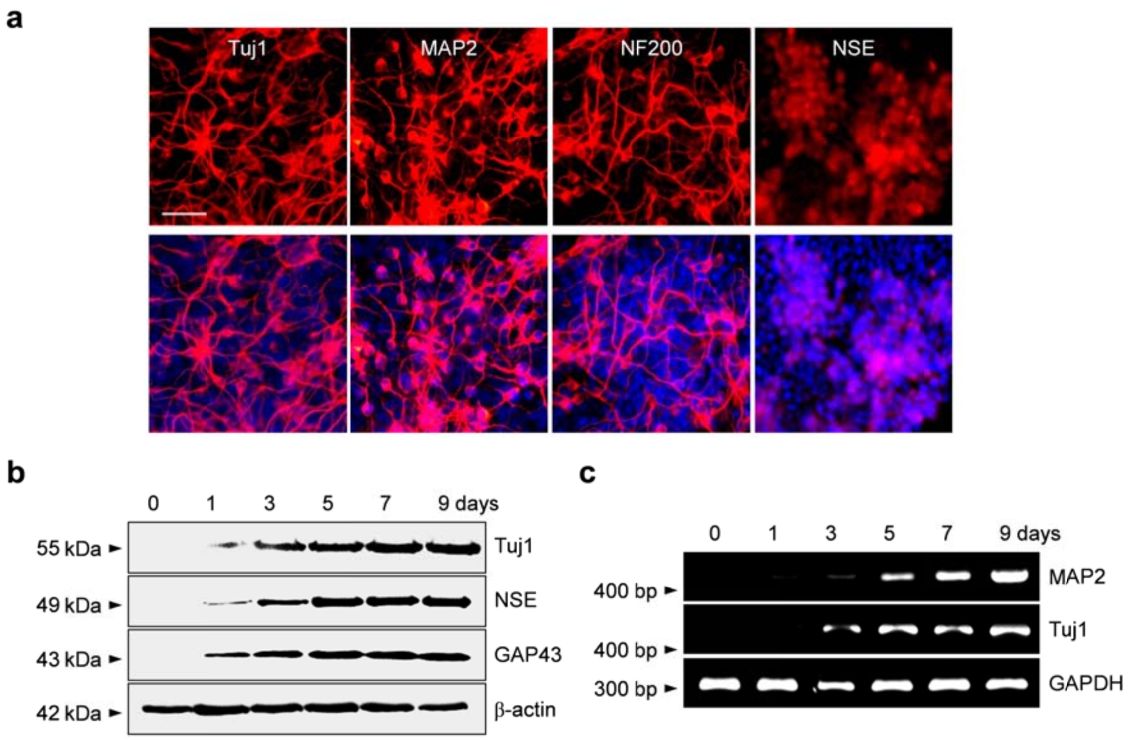

Figure S1. Induction of neuronal differentiation of P19 cells by retinoic acid. (**a**) P19 cells were incubated with 1 μM retinoic acid for 9 days. (Upper panel) The treated cells were immunostained with neuron-specific antibodies. (Lower panel) Merged images of cells treated with antibodies and DAPI (blue). Scale bar: 50 μm. (**b**) Expression levels of neuron-specific markers in the treated P19 cells were examined at various times by using western blot and (**c**) RT-PCR analyses.

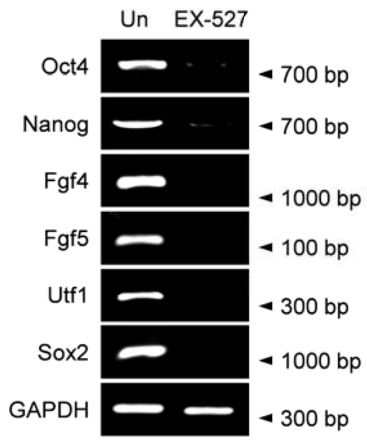

Figure S2. Suppression of stemness of P19 cells by EX-527. P19 cells were incubated with 100 μM EX-527 for 9 days. Expression levels of stemness-related genes were measured by using RT-PCR analysis. Stemness-related genes were downregulated in the treated cells. 'Un' indicates no treatment of P19 cells with EX-527.

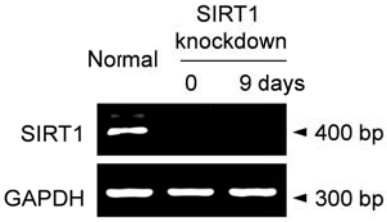

Figure S3. Knockdown of SIRT1 in P19 cells. A SIRT1 gene was not expressed in P19 cells by shRNA-mediated knockdown of SIRT1.

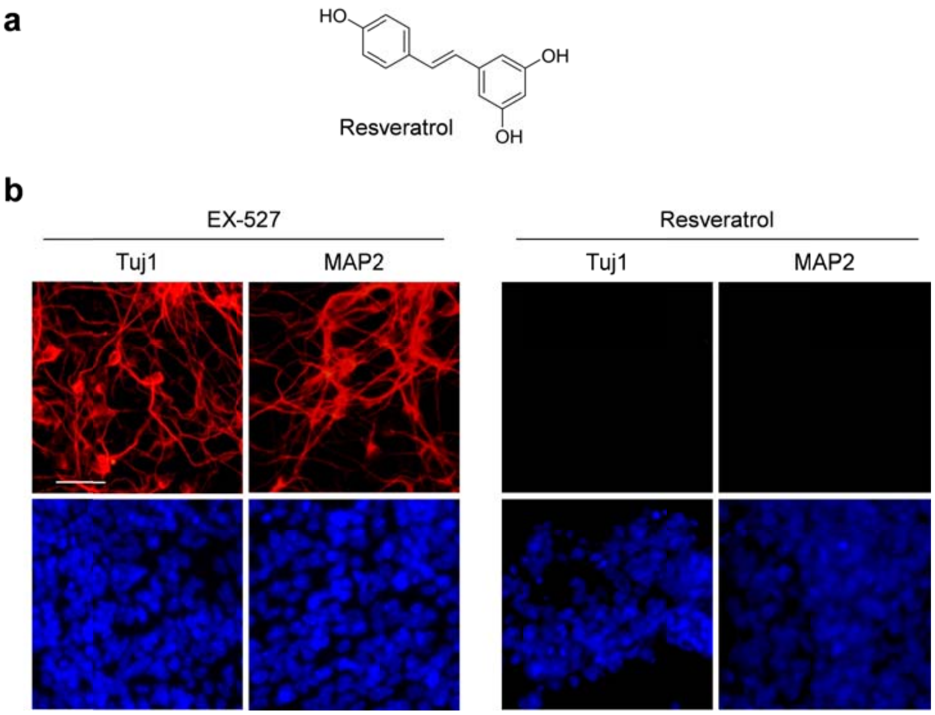

Figure S4. A SIRT1 activator (resveratrol) does not promote neurogenesis. (**a**) Chemical structure of resveratrol. (**b**) P19 cells were incubated with resveratrol or EX-527 for 9 days. (Upper panel) The treated cells were immunostained with neuron-specific antibodies. (Bottom panel) The nucleus of the treated cells was stained with DAPI. Scale bar, 50 μm.

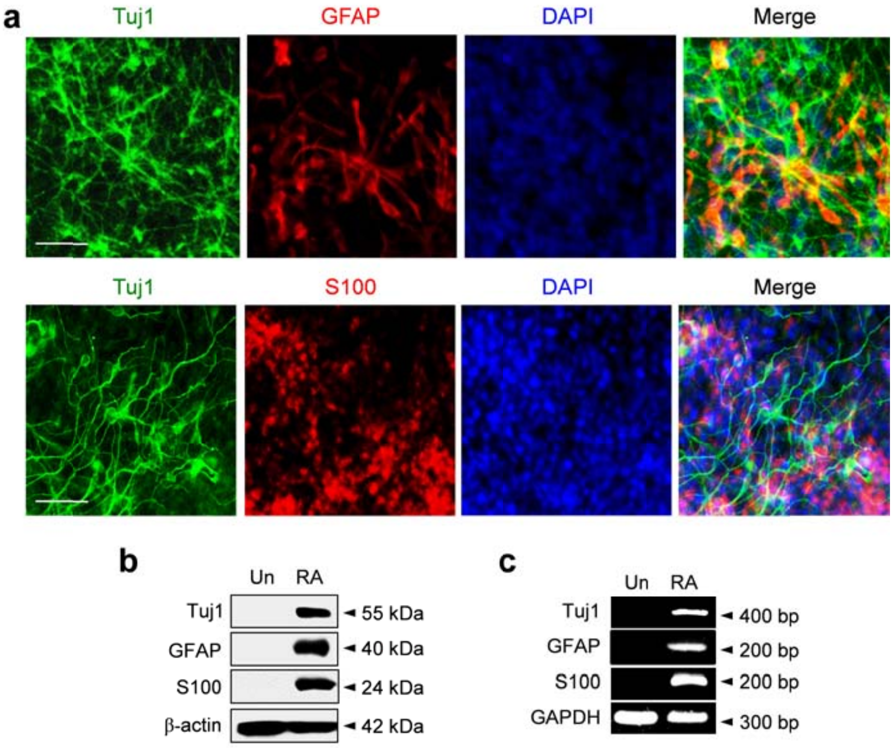

Figure S5. Retinoic acid induces differentiation of P19 cells into neurons and astrocytes. (**a**) P19 cells were incubated with 1 μM retinoic acid (RA) for 9 days. The cells were immunostained with antibodies against neuronal (Tuj1, green) and astrocyte-specific markers (upper: GFAP and lower: S100, red). The nucleus of cells was stained with DAPI. Scale bar, 50 μm. (**b**) P19 cells were incubated with 1 μM retinoic acid for 9 days. The expression levels of neuronal and astrocyte-specific markers in the treated cells were determined by using western blot and (**c**) RT-PCR analyses. 'Un' indicates no treatment of P19 cells with retinoic acid.

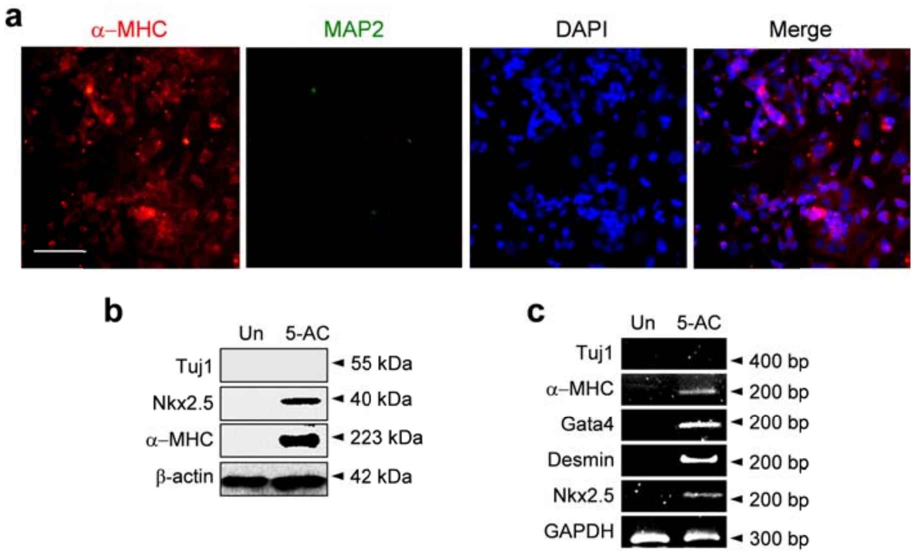

Figure S6. P19 cells are differentiated into cardiac cells by treatment with 5-azacytidine. (**a**) P19 cells were treated with 1 μM 5-azacytidine (5-AC) for 14 days. The cells were immunostained with antibodies against neuronal (MAP2, green) and cardiac markers (α-MHC, red). The nucleus of the cells was stained with DAPI. Scale bar, 50 μm. (**b**) P19 cells were treated with 1 μM 5-azacytidine for 14 days. Expression levels of neuronal and cardiac markers in the treated cells were examined by using western blot and (**c**) RT-PCR analyses. 'Un' indicates no treatment of P19 cells with 5-azacytidine.

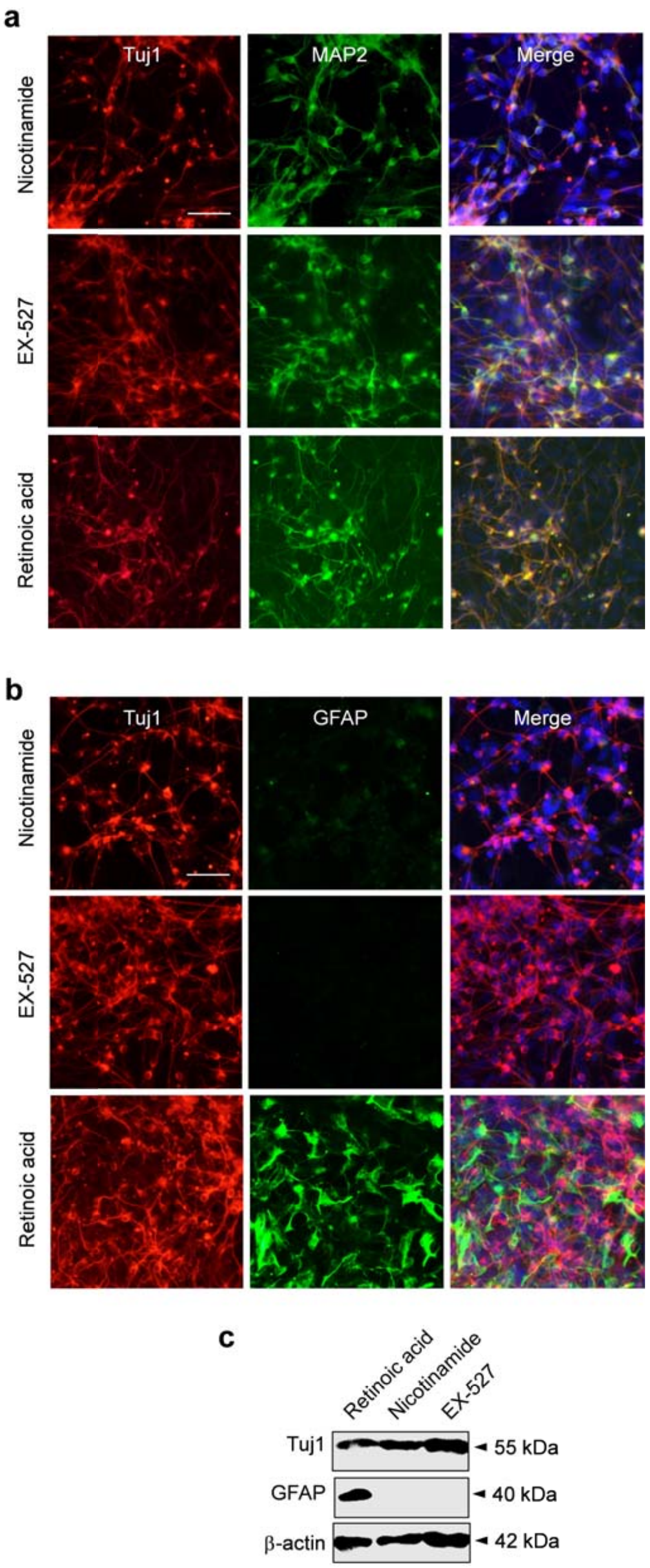

Figure S7. Differentiation of P19 cells induced by treatment with nicotinamide, EX-527 and retinoic acid. (**a**) P19 cells were incubated with 10 mM nicotinamide, 100 μM EX-527

or 1 μM retinoic acid for 9 days. The cells were immunostained with antibodies against neuronal markers. Merged images include DAPI to illustrate nuclei. Scale bar, 50 μm. (**b**) P19 cells were incubated with 10 mM nicotinamide, 100 μM EX-527 or 1 μM retinoic acid for 14 days. The cells were immunostained with antibodies against neuronal (Tuj1, red) and astrocyte-specific markers (GFAP, green). Merged images include DAPI to illustrate nuclei. Scale bar, 50 μm. (**c**) P19 cells were treated with 10 mM nicotinamide, 100 μM EX-527 or 1 μM retinoic acid for 14 days. Expression levels of neuronal and astrocyte-specific markers in the treated cells were determined by using western blot analysis.

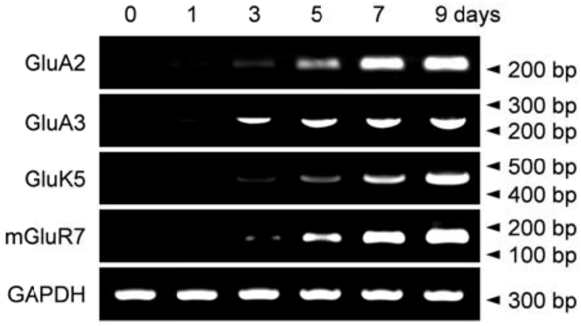

Figure S8. P19 cells treated with EX-527 express glutamate receptor genes. P19 cells were incubated with 100 μM EX-527 for the indicated times. Expression levels of glutamate receptor genes in the treated cells were examined by using RT-PCR analysis.

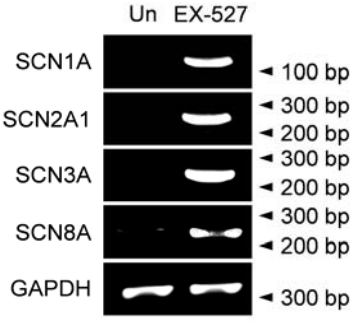

Figure S9. P19 cells treated with EX-527 express voltage-gated sodium channel transcripts. P19 cells were incubated with 100 μM EX-527 for 9 days. Expression levels of sodium channel transcripts in the treated cells were examined by using RT-PCR analysis. 'Un' indicates no treatment of P19 cells with EX-527.
